# Road traffic accidents, still a challenge in South Africa

**DOI:** 10.4102/safp.v67i1.6104

**Published:** 2025-06-02

**Authors:** Indiran P. Govender, David K.K. Masanabo

**Affiliations:** 1 Department Family Medicine and Primary Health Care, Faculty of Health Sciences, Sefako Makgatho Healthcare Sciences University, Pretoria, South Africa; 2 School of Medicine, Faculty of Health Sciences, Sefako Makgatho Healthcare Sciences University, Pretoria, South Africa; 3 Dr George Mukhari Academic Hospital, Pretoria, South Africa

As we are nearing the end of another year, we look back and reflect on our achievements, which usually fills us with a sense of joy and happiness. We also reflect on our difficulties, with a purpose of coming up with measures to overcome them. However, this time of the year also presents us with increased road traffic accidents (RTAs). This is one of the greatest challenges that South Africa (and the world) faces. According to the World Health Organization (WHO), RTA cause deaths of approximately 1.19 million people globally each year.^1^ It often involves various modes of transport such as cars, buses, motorbikes, bicycles, trucks and pedestrians.^2^ Road traffic accident is among the top 10 causes of death worldwide and a leading cause of mortality in young people (5–29 years of age), with approximately 93% of deaths occurring in low- and middle-income countries.^2,3^ Road traffic accidents are serious public health problems with a current global fatality rate of 16.4 per 100 000.^2^ This shows the extent of the problem both nationally and internationally, and it continues to cause a significant rise in morbidity and mortality.

Research studies reported the RTA frequency (the number of accidents that occur per year, typically over a 3–5-year period, on a specific road section or intersection)^4^ to be more than 20%, which is disturbing given numerous policies and programmes in place for road safety and reducing RTA in different countries. Studies carried out in various countries reported RTA frequency of 63.6% in Brazil,^5^ 30 % in Vietnam,^6^ 41.4% in India^7^ and 51.50% in Iran.^8^

In developing countries, the RTA frequency was found to be higher than in developed countries. In Ghana, the reported RTA frequency was 74.0%, in Nigeria was 87.5%^9^ and in Ethiopia it varied from 23.5% to 62.5%.^10^ This shows that current RTA preventative measures put in place in various countries are still inadequate to address this challenge and decrease RTA mortality.

South Africa is no exception to this staggering rise in RTA. It is one of the major public health problems we face with a tremendous burden on the already strained healthcare system. It was reported that 12 436 people lost their lives because of RTAs in the year 2022, with a population death rate of 20.7 per 100 000 population.^11,12^ In 2023, there was 10 180 fatal crashes that led to 11 883 deaths and 5360 pedestrians losing their lives because of RTA. It is scary to observe that over 21% of RTA mortality in 2023 involved hit-and-run incidents, meaning the perpetrator may not be apprehended to face his or her crime.^13^ The coronavirus disease 2019 (COVID-19) pandemic with curfew and reduced access to alcohol showed us that drastic reduction of road traffic accidents is possible. The number of deaths as a result of road traffic accidents decreased significantly drastically during this period.

There are many causes of RTA, and often there are multiple factors in play during one RTA. The factors contributing most to RTA in South Africa include speeding, distraction, use of alcohol or other substances, inadequate law enforcement, use of unroadworthy vehicles, poor road conditions, pedestrians’ lack of knowledge, etc. We will discuss the common causes.

According to the Western Cape road safety facts^14^, the speed limits in South Africa are: 60 km/h on a public road within an urban area, 100 km/h on public roads outside an urban area, which is not a freeway and 120 km/h on freeways.^15^ A motor vehicle moving at a high speed has an increased risk of causing an accident and causing severe injuries to other road users, such as pedestrians. The ability to avert an accident is reduced while driving in high speed.^15^

Distraction among road users has been found to be a growing problem that contributes significantly to RTA.^15^ Types of distraction can be visual distraction (e.g., looking away from the road, texting on a cell phone, etc.), auditory distraction (e.g. responding to a ringing cell phone), biomechanical distraction (e.g. manually adjusting the radio volume) and cognitive distraction (e.g. being lost in thought).^16^ This problem is not unique to the drivers, pedestrians, cyclists and riders also experience distraction, which poses a risk of a traffic collision.^15^

Driving under the influence of alcohol and/or any other psychoactive substance has been found to increases the risk of RTA.^15^ To be precise, drunk driving has been found to claim lives every 50 min and about 10 000 lives are lost every year.^17^ Alcohol leads to RTA by impairing the essential abilities to operate a motor vehicle safely as well decision making to avoid an accident.^15^ The risk of RTA starts at low levels of blood alcohol concentration (BAC) and peaks when the BAC is ≥ 0.04 g/dL.^18^ One study reported that the alcohol-related RTA statistics may actually be more that reported.^15^

Preventative measures that can be employed to reduce and prevent RTA in South Africa are increased visibility of traffic police officials on the road, and this will ensure most of the road users obey the rules of the road. Road users tend to modify their behaviour on the road when they see law enforcement officers.^15^ This is particularly important because human error has been found to be the main cause of accidents and crashes on the roads.^15^ Empowering traffic officers to have in-depth knowledge, skills and attitude about road safety is also an important preventative measure. Unethical behaviours such as traffic officials soliciting bribes from road users should be addressed harshly, as RTA stats may continue to rise from such behaviours.

The use of technology in the prevention of RTA is also an important measure to employ. Various devices such as breathalyser testing equipment to detect the blood alcohol limit, speed cameras, surveillance cameras, body worn cameras, etc. can help the road users to adhere to the rules of the road and thus bring down the number of RTA.^15^ Road traffic users may consider some of the newer technologies such as card dash cameras or fleet dash cameras to support and enhance safe driving, thus minimising the risk of RTA.^15^

The basic rule of the road is that the road users should keep their eyes on the road, hands on the wheel and the mind concentrating on driving. One way to achieve this is by minimising or avoiding distractions while on the road. Education programmes and awareness campaigns on how to avoid distractions among road users are essential; this will include hands free operation of devices like cell phones, no texting while driving, etc. These programmes should be offered to all road users but especially young and new drivers, as they are more at risk of RTA.^15^

Learning road traffic safety and driving safely on the roads may even be started at high school. The road traffic laws in South Arica must be strictly enforced and bribery and corruption need to be weeded out. At present it seems the traffic laws are blatantly broken with no serious consequences. These laws are likened to guidelines rather than enforceable laws of our country.

Family physicians can play a vital role by leading health awareness campaigns on the prevention of RTA. Such campaigns have been found to have a positive effect on the prevention of RTA and RTI (road traffic injuries).^19^ As frontline and first contact healthcare practitioners, we can promote good health and prevent RTA by talking to patients about safety on the roads such as not driving under influence of alcohol, the harmful effects of alcohol, importance of wearing seat belts and not texting while driving, et cetera. These campaigns should be held both inside and outside the healthcare facilities, in schools, places of worship, community gatherings, etc. This will help to spread the message to as many members of the community as possible to ensure the effectiveness of these programmes.

We need to continue to see ourselves as a network of support services such as referring people with alcohol abuse problems for psychological support. As family physicians responsible for holistic healthcare, it is our duty to play an active role in decreasing the carnage on our roads.

## References

[1] WHO. Road traffic injuries [homepage on the Internet]. 2024 [cited 2024 Nov 11]. Available from: https://www.who.int/health-topics/road-safety#tab=tab_1

[2] Hareru HE, Negassa B, Kassa Abebe R, et al. The epidemiology of road traffic accidents and associated factors among drivers in Dilla Town, Southern Ethiopia. Front Public Health. 2022;10:1007308. 10.3389/fpubh.2022.1007308 36438205 PMC9686279

[3] World Health Organization. Road traffic injuries [homepage on the Internet]. [cited 2019 Jul 14]. Available from: https://www.who.int/news-room/fact-sheets/detail/road-traffic-injuries

[4] Almeida GC, De Medeiros FDCD, Pinto LO, De Oliveira Moura JMB, Lima KC. Prevalence and factors associated with traffic accidents involving motorcycle taxis. Rev Bras Enferm. 2016;69(2):382–388. 10.1590/0034-7167.2016690223i 27280576

[5] Nguyen-Phuoc DQ, Nguyen HA, De Gruyter C, Su DN, Nguyen VH. Exploring the prevalence and factors associated with self-reported traffic crashes among app-based motorcycle taxis in Vietnam. Transp Policy. 2019;81:68–74. 10.1016/j.tranpol.2019.06.006

[6] Ashish T, Devang R. Prevalence of road traffic accidents and driving practices among young drivers. Healthline [serial online]. 2011 [cited 2024 Nov 11]; 2(2):72–75. Available from: http://www.iapsmgc.org/OA17V2I2.pdf

[7] Najimi-Varzaneh A, Gholami Fesharaki M. Prevalence of road traffic accidents in Iran: A systematic review, GIS and meta-analysis. Iran Red Crescent Med J. 2018;20(10):e83852. 10.5812/ircmj.83852

[8] Konlan KD, Doat AR, Mohammed I, et al. Prevalence and pattern of road traffic accidents among commercial motorcyclists in the Central Tongu District, Ghana. Sci World J. 2020;2020(1):3718. 10.1155/2020/9493718 PMC7285403 32565754

[9] Johnson OE. Prevalence and pattern of road traffic accidents among commercial motorcyclists in a city in Southern Nigeria. Educ Res [serial online]. 2012 [cited 2024 Nov 16];3(6):537–542. Available from: http://www.interesjournals.org/ER

[10] Asefa NG, Ingale L, Shumey A, Yang H. Prevalence and factors associated with road traffic crash among taxi drivers in Mekelle Town, Northern Ethiopia, 2014: A cross sectional study. PLoS One. 2015;10(3):e0118675. 10.1371/journal.pone.0118675 25781940 PMC4363695

[11] Sinclair M. The promotion of road safety by healthcare professionals in South Africa. S Afr Med J. 2013;103(9):614–615. 10.7196/samj.7335 24300676

[12] Geduld H, Sinclair M, Steyn E, Chu K. Road traffic injuries in South Africa: A complex global health crisis. Ann Glob Health. 2024;90(1):26, 1–3. 10.5334/aogh.4249 38618273 PMC11012091

[13] Festive season road safety campaign remarks by transport minister Ms Barbara Creecy [homepage on the Internet]. 2024 [cited 2024 Nov 21]. Available from: https://www.transport.gov.za>2023/02

[14] Road safety facts. Western Cape government. [cited 2025 Jan 20]. Available from: https://d7.westerncape.gov.za/general-publication/road-safety-facts

[15] Mmakwena M. Road traffic accident in South Africa: Challenges and solutions. Int J Res Bus Soc Sci. 2023;12(8):557–565. 10.20525/ijrbs.v12i8.2940

[16] Chan M. Distracted road use: A literature review [homepage on the Internet]. 2016 [cited 2024 Nov 21]. Available from: https://www.edmonton.ca/public-files/assets/document?path=RoadsTraffic/DistractedRoadUseReport

[17] Mattar W. How does drunk driving cause accidents? [homepage on the Internet]. 2021 [cited 2024 Nov 21]. Available from: https://williammatar.com/blog/drunk-driving/how-does-drunk-driving-cause-accidents/?utm_content=organic_direct

[18] World Health Organization. Decade of action for road safety [homepage on the Internet]. 2023 [cited 2023 Jul 31]. Available from: https://www.who.int/news-room/fact-sheet/detail/road-traffic-injuries

[19] Van Schagen I, Machata K. Best practices in road safety: Handbook for measures at the country level. Luxembourg: Publications Office of the European Communities Eur-OP; 2010.

